# A Pathological Complete Response After Pembrolizumab for DNA Mismatch Repair-Deficient/Microsatellite Instability-High Colorectal Cancer Enabling Curative Surgery: A Case Report

**DOI:** 10.7759/cureus.100109

**Published:** 2025-12-26

**Authors:** Bernard L Hanekom, Andrew Coveney

**Affiliations:** 1 Faculty of Medicine, University of Western Australia, Perth, AUS; 2 General Surgery, Sir Charles Gairdner Hospital, Perth, AUS

**Keywords:** colorectal cancer surgery, complete pathological response, general surgery, mismatch repair deficiency, pembrolizumab

## Abstract

We present the case of a 49-year-old man with initially unresectable, metastatic mismatch repair-deficient colorectal cancer (CRC) who achieved a complete pathological response following neoadjuvant immunotherapy. Metastatic CRC is rarely curable, and complete tumour eradication with standard chemotherapy is exceedingly uncommon. However, deficient mismatch repair/ microsatellite instability-high (dMMR/MSI-H) tumours are uniquely sensitive to immune checkpoint blockade. This patient presented with sigmoid adenocarcinoma with extensive nodal and liver metastatic disease and underwent treatment with pembrolizumab. Restaging imaging demonstrated tumour regression; however, his treatment course was complicated by recurrent small bowel obstructions and the development of a pelvic abscess at the primary cancer site, necessitating interruption of systemic treatment. Multidisciplinary consensus recommended that he should undergo salvage surgical resection. Postoperative pathology surprisingly demonstrated no viable cancer cells in the resected colon, lymph nodes, or hepatic tissue (ypT0N0), confirming a complete pathological response to immunotherapy. Pembrolizumab was resumed postoperatively, and at 10 months post resection, the patient remains disease-free clinically and radiologically. This case demonstrates the potential for PD-1 inhibitor therapy to induce profound remission even in metastatic CRC, potentially enabling long-term disease control. It underscores the importance of routine mismatch repair (MMR) status testing to identify patients who may benefit from immunotherapy and highlights management considerations in patients with exceptional treatment response. This remarkable outcome adds to the growing evidence that a subset of advanced CRC patients can achieve durable remission or even cure with immunotherapy, signalling a shift in the treatment paradigm of these dMMR/MSI-H metastatic CRC.

## Introduction

Colorectal cancer (CRC) is a leading cause of cancer mortality, and up to 20% of patients present with metastatic disease, which is associated with a significantly poorer prognosis [1]. While systemic chemotherapy has improved median overall survival to around 30 months in metastatic CRC (mCRC), long-term cure remains rare [1,2]. A small subset of CRC with deficient DNA mismatch repair (dMMR) and high microsatellite instability (MSI-H) is highly responsive to immune checkpoint inhibitor therapy [3]. These dMMR/MSI-H tumours constitute approximately 4-5% of metastatic CRC [3] and harbour high mutational burdens and neoantigen loads, rendering them remarkably immunogenic and susceptible to PD-1 blockade [3,4].

Even with aggressive chemotherapy, survival for mCRC is poor, and pathological complete response (pCR) in mCRC is rare [1]. By contrast, with the advent of immunotherapy, outcomes for a subset of dMMR/MSI-H mCRC have drastically improved in recent years [2,4]. Landmark trials have demonstrated the superior efficacy of immunotherapy compared to standard chemotherapy in the management of these patients with metastatic disease. The KEYNOTE-177 trial comparing pembrolizumab to standard chemotherapy demonstrated that pembrolizumab had significantly prolonged progression-free survival and achieved a complete radiographic response in 11% of patients vs only 4% with standard chemotherapy [5]. The CHECKMATE trial compared immunotherapy with nivolumab and ipilimumab to chemotherapy and demonstrated prolonged progression-free survival with immunotherapy compared to standard chemotherapy [6]. The GARNET trial evaluated the efficacy and safety of dostarlimab in managing dMMR/MSI-H solid tumours. The trial reported durable treatment responses, including objective response rates of 44% and a median progression-free survival of 8.4 months in the colorectal cancer subgroup, similar to other immunotherapy options [7]. The remarkable sensitivity of dMMR/MSI-H mCRC to immunotherapy has established immunotherapy drugs such as pembrolizumab as first-line therapy options within this population [3].

Here, we present a patient with dMMR/MSI-H mCRC who, following neoadjuvant immunotherapy with pembrolizumab, was found to have pCR at the time of surgery. The case highlights the dramatic ability of immunotherapy at downstaging these tumours and raises important clinical questions, including the role of surgical intervention after immunotherapy and the role of adjuvant therapy after pCR.

## Case presentation

**Figure 1:**
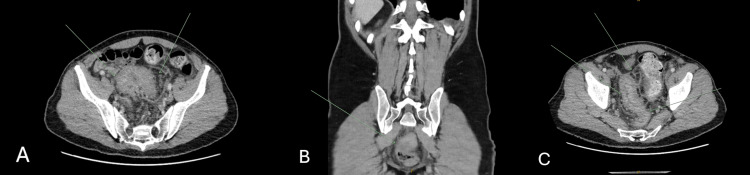
CT slices demonstrating rectosigmoid colitis on axial (A,C) and coronal (B) views

A 49-year-old male patient with previous heavy smoking (35 pack-years) and intravenous drug use presented with several months of altered bowel habits. He reported symptoms of worsening constipation alternating with frequent small-calibre stools, intermittent rectal bleeding, abdominal pain, and weight loss. There was no known personal or family history of CRC or other Lynch syndrome-associated cancers, and he had no significant comorbidities. An initial computed tomography (CT) scan of the abdomen and pelvis showed inflammatory changes suggesting rectosigmoid colitis without a distinct mass identified (Figure 1).

On follow-up, the patient reported persistent symptoms, and physical examination revealed mild left lower quadrant tenderness without a palpable mass. Colonoscopy was performed, which demonstrated a near-obstructing circumferential, malignant-appearing mass in the mid to distal sigmoid colon. The colonoscope could not pass beyond the mass, which was friable, ulcerated, and displayed contact bleeding. Biopsies were taken and sent for histopathology.

**Figure 2:**
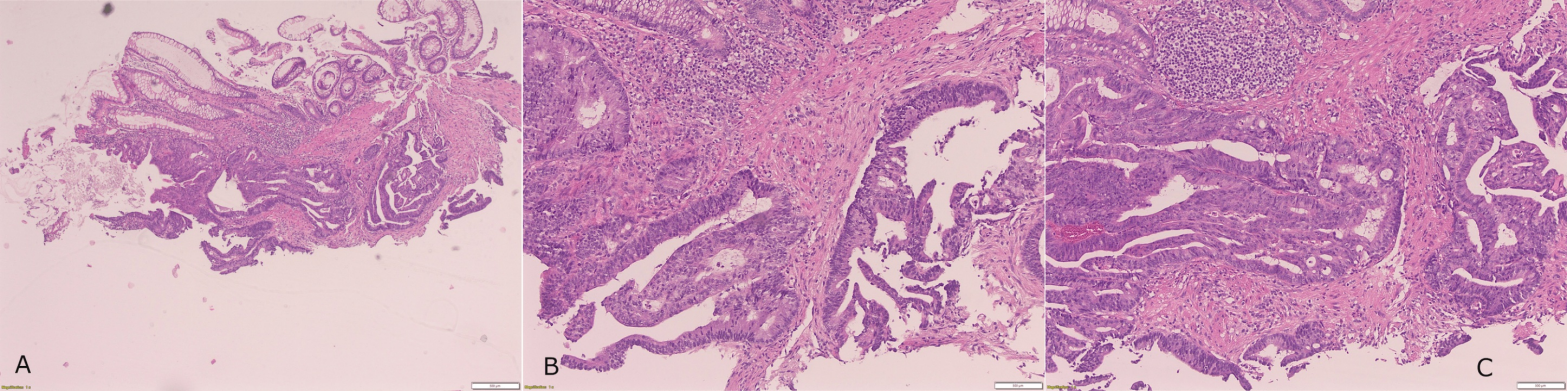
Histology slides with H&E staining at 4x (A) and 10x (B,C) magnification

**Figure 3:**
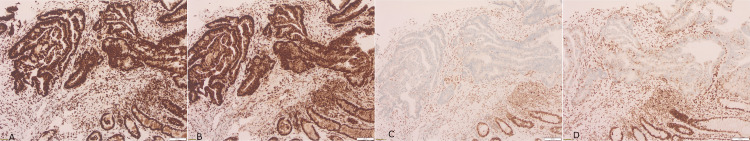
Immunohistochemistry slides of MLH1 (A), PMS2 (B), MSH2 (C), and MSH6 (D)

Microscopy showed high-grade dysplastic glandular epithelium with cribriform and fused glands showing superficial invasion of the lamina propria, suggestive of well-differentiated adenocarcinoma (Figure 2). There was no lymphovascular invasion or perineural tumour infiltration seen. Immunohistochemistry with immunoperoxidase staining showed retained expression of MLH-1 and PMS-2 but loss of expression of MSH2 and MSH6, indicating mismatch repair deficiency. Molecular pathology studies performed identified the presence of *KRAS* G12D mutation, as well as no mutations in *BRAF*, *NRAS*, *PIK3CA*, *PTEN*, and *AKT1* (Figure 3).

**Figure 4:**
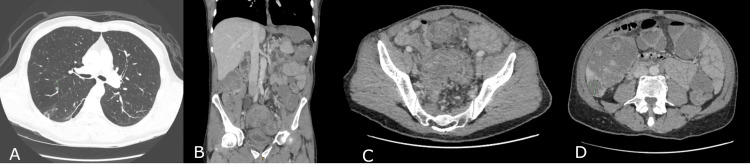
CT imaging with (A) axial CT slice demonstrating lung lesion, (B) coronal CT slice demonstrating sigmoid mass, (C) axial slice demonstrating sigmoid mass and (D) axial slice demonstrating hepatic metastasis

Repeat staging with a CT chest/abdomen/pelvis (Figure 4) showed a long segment sigmoid colon tumour with thickening and luminal narrowing, multiple enlarged locoregional lymph nodes, numerous bilobar hepatic metastases (the largest measuring 3.2 cm), and a suspicious 6 mm pulmonary nodule within the right upper lobe. The primary tumour appeared to invade adjacent organs, including the bladder and left pelvic sidewall.

The patient was admitted emergently for operative management of an impending large bowel obstruction and underwent a laparotomy and formation of a loop sigmoid colostomy as a palliative measure to enable him to undergo systemic treatment. Intraoperatively, the tumour was confirmed to be densely adherent to adjacent structures, including the bladder, pelvic sidewall, and retroperitoneum, confirming locally advanced, unresectable disease.

Given the dMMR/MSI-H tumour status, he was initiated on immunotherapy with pembrolizumab given as 200 mg IV every three weeks. Repeat staging two months after starting immunotherapy demonstrated partial response in the abdomen and pelvic disease, a reduction in regional lymphadenopathy, unchanged hepatic disease, and an unchanged pulmonary nodule. His immunotherapy course was complicated by recurrent episodes of small bowel obstruction, which were managed conservatively. The patient additionally developed right-sided obstructive uropathy during his immunotherapy course, which was managed successfully with rigid cystoscopy and placement of a right ureteric JJ stent.

**Figure 5:**
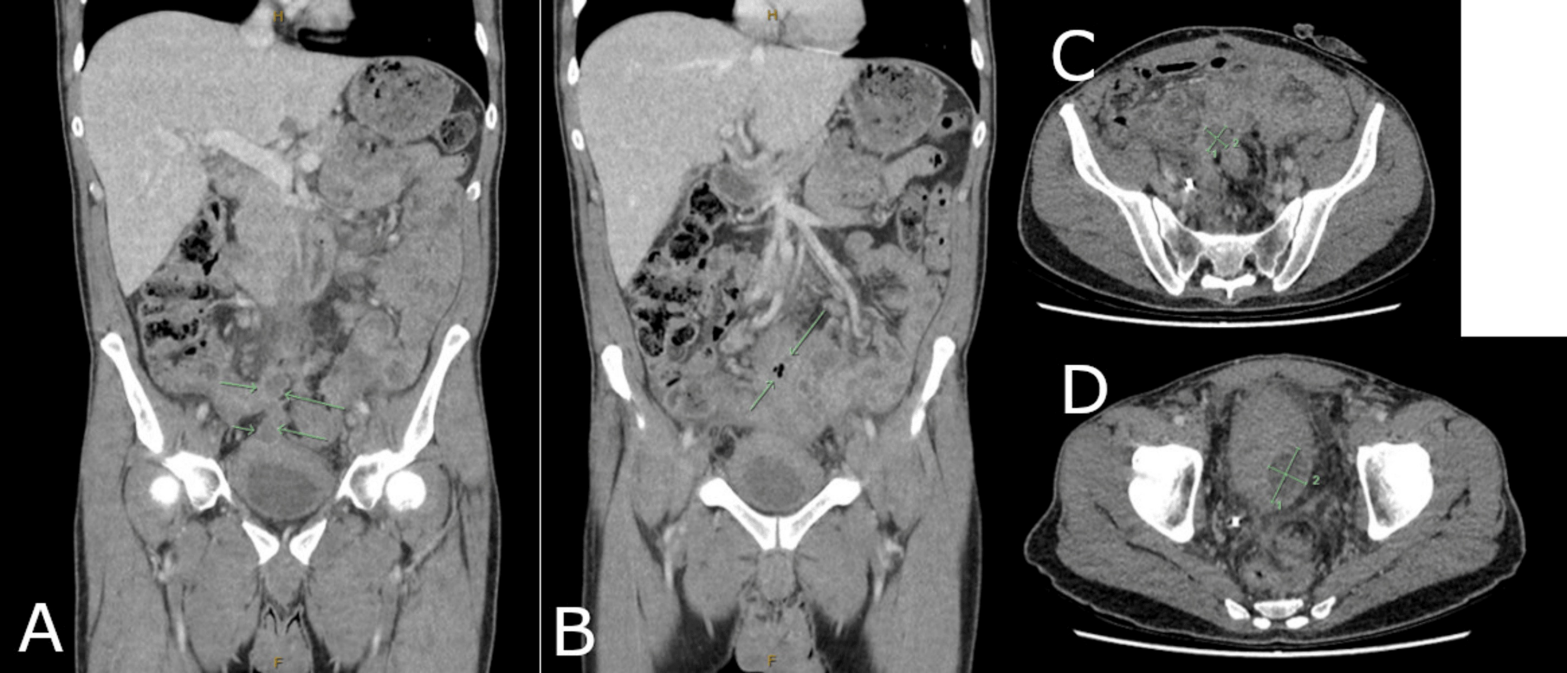
CT images demonstrating pelvic abscess on coronal slices (A,B), and axial slices (C,D)

Four months after initiating immunotherapy, the patient presented with high fevers and pelvic pain and underwent a repeat CT, which demonstrated a new and multiloculated pelvic abscess within the region of his tumour, along with extensive inflammatory change and pockets of gas and fluid. (Figure 5). He was managed as an inpatient with radiologically guided drainage and antibiotics. His overall condition improved during his admission, but in the setting of ongoing pelvic sepsis, his immunotherapy was interrupted.

Multidisciplinary consensus recommended proceeding with operative management to attempt salvage surgical resection of any residual pelvic disease and control the pelvic sepsis. He underwent a laparotomy along with an ultra-low anterior resection (ULAR) and en-bloc resection of involved small bowel and ileocolic bowel, along with a wedge resection of macroscopic liver disease and a reversal of his colostomy. His postoperative course was uncomplicated, and he was discharged from the hospital on day 10 postoperatively.

Postoperative histopathology surprisingly returned a complete pathological response to treatment. The ULAR specimen of the rectosigmoid contained only fibrosis, inflammatory cells, and treatment-related changes with no viable adenocarcinoma identified. Fifty regional lymph nodes from the mesorectal and mesocolic resections were all negative for malignancy. Small bowel and ileocolic specimens showed no cancer, and the wedge-resected liver tissue showed only necrosis and fibrous scar without residual metastatic tumour. In summary, this confirmed a pCR (ypT0 N0) to neoadjuvant immunotherapy in both the primary and metastatic sites.

Subsequent multidisciplinary team (MDT) recommended that he resume pembrolizumab and complete two years of immunotherapy. At 10 months post resection, the patient remains free of clinical or radiological signs of recurrence. A recent whole-body fluorodeoxyglucose (FDG) PET scan showed no metabolically active disease, and he remains on pembrolizumab therapy without further immune-related complications. His primary postoperative challenge has been bowel dysfunction consistent with low anterior resection syndrome, which is being managed conservatively. He remains on close clinical review with tumour markers and periodic imaging. The patient reports a good quality of life and gratitude towards the overall positive outcome.

## Discussion

This case demonstrates the profound efficacy of immunotherapy in dMMR/MSI-H mCRC. Historically, mCRC is managed with palliative intent, and curative outcomes are typically rare [1]. There is emerging evidence that immunotherapy can induce complete responses in this subset of mCRC, for example, the KEYNOTE trial identified a complete radiological response of 11% in mCRC treated with pembrolizumab [5]. Several cases report pCR in patients with dMMR/MSI-H mCRC. Igaue et al. present a case of dMMR/MSI-H mCRC in which the patient underwent immunotherapy with nivolumab plus ipilimumab after progression on chemotherapy; immunotherapy resulted in tumour downstaging, and subsequent surgical resection demonstrated pCR [8]. Xu et al. report a case of complete radiologic response of dMMR/MSI-H mCRC in a patient treated with pembrolizumab, who had similarly previously progressed rapidly on chemotherapy [9].

Lee et al. presented a patient with dMMR/MSI-H mCRC who was managed first-line with pembrolizumab and achieved a complete radiological response [10]. At surgery, the colon was noted to have spontaneously transected at the tumour site, presumably from immune-mediated tissue remodelling, and histopathology subsequently returned a pCR (ypT0N0). In our case, the development of a pelvic abscess during immunotherapy may represent a similar phenomenon of rapid tumour death resulting in tumour necrosis, leading to subsequent abscess formation. Importantly, in this case, complications during immunotherapy required multidisciplinary input but fortunately did not prevent a successful outcome. As illustrated in this case, in the era of immunotherapy for dMMR/MSI-H mCRC, downstaging of metastatic disease to a point of resectability may become more commonplace with dramatic tumour regression or even eradication preoperatively.

An important discussion point from this case has been the optimal management after complete response to systemic therapy. Specifically, the role of immunotherapy after surgery in a patient with pCR remains unclear. The outcome from our MDT discussion recommended resuming pembrolizumab to consolidate the treatment response. Lack of clear guidelines in this postoperative period highlights the need for ongoing research on how best to manage patients who achieve a complete response with systemic therapy.

Finally, this case emphasizes the importance of comprehensive molecular testing and a well-coordinated multidisciplinary approach in the management of advanced CRC. The patient’s tumour was promptly identified as dMMR/MSI-H status, enabling eligibility for immunotherapy. This likely altered the trajectory of his disease from an almost certain terminal outcome to a chance for a cure. Equally important was the coordination within the MDT to manage various complications and to agree on optimal timing for surgical intervention. In summary, the interplay of advanced diagnostics, novel systemic therapy, and multidisciplinary care in this case led to an exceptional outcome that would have historically been unattainable in mCRC.

## Conclusions

We reported a unique case of a 49-year-old man with initially unresectable, metastatic dMMR/MSI-H CRC who achieved a pCR following neoadjuvant immunotherapy with pembrolizumab and salvage surgical resection. This outcome highlights the positive outcomes from immune checkpoint inhibitors for a subset of mCRC patients with dMMR/MSI-H status that have historically had a dismal prognosis. 

Key lessons from this case include the value of routine dMMR/MSI-H testing in CRC, which can identify patients who are likely to benefit from highly effective immunotherapy, as well as the importance of a multidisciplinary approach to managing these cases. As immunotherapy becomes integrated into earlier lines of therapy, clinicians will increasingly encounter scenarios of complete response and face decisions regarding the role of surgery and duration of therapy. This case adds to the growing body of evidence that some patients with advanced CRC can achieve long-term remission or cure with immunotherapy, marking a potential shift in management for this subgroup of patients.
